# Impact of Rhegmatogenous Retinal Detachment on Macular Vascular and Functional Integrity

**DOI:** 10.3390/biomedicines12122911

**Published:** 2024-12-20

**Authors:** María Dolores Díaz-Barreda, Ana Boned-Murillo, Isabel Bartolomé-Sesé, María Sopeña-Pinilla, Elvira Orduna-Hospital, Guisela Fernández-Espinosa, Isabel Pinilla

**Affiliations:** 1 Department of Ophthalmology, Virgen de la Luz Hospital, 16002 Cuenca, Spain; mddiaz@salud.aragon.es; 2 Aragón Health Research Institute (IIS Aragón), 50009 Zaragoza, Spain; anabomu@hotmail.com (A.B.-M.); issbartolome@gmail.com (I.B.-S.); eordunahospital@unizar.es (E.O.-H.); guisela.fernandez3@gmail.com (G.F.-E.); 3 Fundación de Oftalmología Médica de la Comunitat Valenciana, 46015 Valencia, Spain; 4 Department of Ophthalmology, Lozano Blesa University Hospital, 50009 Zaragoza, Spain; 5 Department of Ophthalmology, Miguel Servet University Hospital, 50009 Zaragoza, Spain; 6 Department of Applied Physics, University of Zaragoza, 50009 Zaragoza, Spain; 7 Department of Surgery, University of Zaragoza, 50009 Zaragoza, Spain

**Keywords:** rhegmatogenous retinal detachment, optical coherence tomography angiography, OCTA, microperimetry, MAIA, surgery, correlation

## Abstract

Objectives: This study aimed to evaluate the correlations between optical coherence tomography angiography (OCTA), best corrected visual acuity (BCVA), and macular integrity assessment (MAIA) microperimetry (MP) in both a control group and patients with rhegmatogenous retinal detachment (RRD). Additionally, it assessed differences between the groups and examined whether the time from symptom onset to surgery influenced microvascular or functional changes in the RRD group. Methods: A cross-sectional study was conducted involving 47 patients who had undergone successful RRD surgery with pars plana vitrectomy (PPV) and sulfur-hexafluoride (SF6) gas injection, with or without scleral buckling (SB), and a control group of 136 healthy eyes. All participants underwent comprehensive ophthalmologic examinations, including BCVA, OCTA, and MAIA. In the RRD group, additional data on symptom duration, time from symptom onset to surgery, and time from surgery to testing were collected. Results: The RRD group exhibited significantly worse BCVA (*p* < 0.001) compared to the control group. Significant differences were found in all MAIA sectors, with controls showing superior macular integrity and average threshold values (*p* < 0.001). OCTA analysis revealed differences in the superficial capillary plexus (SCP) and deep capillary plexus (DCP) across various sectors, particularly in the foveal avascular zone (FAZ). In the control group, the vertical diameter of the FAZ in the SCP was positively correlated with most MAIA sectors, while in the DCP, correlations were seen in nearly all sectors. The RRD group showed fewer correlations between OCTA and MAIA, and no significant correlations were found between OCTA parameters and BCVA. However, there were correlations between the time from surgery to testing and MAIA outcomes, indicating improved results with longer intervals. Earlier surgical intervention after symptom onset was associated with better microvascular outcomes. Conclusions: RRD group exhibited significant impairments in BCVA, retinal sensitivity, and microvascular parameters compared to healthy controls. Correlations between OCTA findings and microperimetry were stronger in the control group, whereas the RRD group showed fewer and weaker associations.

## 1. Introduction

Rhegmatogenous retinal detachment (RRD) is a serious condition that compromises retinal integrity and can lead to permanent loss of retinal function. The reported incidence of RRD varies, ranging from 6.3 to 18.2 cases per 100,000 people per year. This incidence is increasing, partly due to population aging and the global rise in myopia. Although anatomical success rate for a single surgery exceeds 85% in many studies, this does not guarantee satisfactory functional recovery [1,2].

Research has extensively explored the gap between anatomical success and functional outcomes in RRD treatment. Various risk factors have been identified that may impact the final best corrected visual acuity (BCVA), including sociodemographic factors, patient-specific characteristics, and factors related to the RRD itself. Furthermore, different therapeutic options have been analyzed to refine the indications for each treatment approach. Technological advances in ophthalmology have significantly contributed to these insights [1,3,4].

Optical coherence tomography angiography (OCTA) has emerged as a valuable tool for evaluating retinal microvascularization, specifically in the superficial capillary plexus (SCP), intermediate capillary plexus (ICP), deep capillary plexus (DCP), and choriocapillaris (CC). By capturing red blood cell movement within retinal vessels, OCTA offers a non-invasive, dye-free, fast, and safe imaging technique [5]. Recent studies have begun documenting changes in microvascularization in eyes that have undergone RRD surgery, with the goal of identifying biomarkers that could predict final BCVA, similar to what has been observed in other pathologies like diabetes mellitus (DM) or retinal vascular occlusions [6,7]. However, BCVA alone is not a good parameter to adequately assess visual functional outcomes [8,9].

In clinical practice, functional tests such as retinal microperimetry (MP) offer a more detailed and objective assessment of retinal function by measuring retinal sensitivity and fixation ability. These tests provide meaningful insights into actual retinal performance [10].

The primary objective of this study was to examine correlations among OCTA metrics, BCVA, and macular integrity assessment (MAIA) MP in both a control group and patients with RRD. As secondary objectives, we analyzed the differences between both groups and investigated whether the time elapsed from symptom onset to surgery influenced microvascular or functional outcomes in the RRD group.

## 2. Materials and Methods

We conducted a cross-sectional, single-center study in the Lozano Blesa University Hospital, Zaragoza, Spain, from June 2022 to June 2023. The study was registered and approved by the Aragon Clinical Research Committee and adhered to the tenets of Helsinki Declaration, as well as Spanish Law 14/2007 on Biomedical Research, Organic Law 3/2018 on the Protection of Personal Data, and Basic Law 41/2002. All participants were over 18 years of age and provided informed consent.

Subjects were divided into two groups. The RRD group consisted of 65 patients with primary RRD who had undergone a single successful surgery (23G pars plana vitrectomy [PPV] with or without scleral buckling [SB] and sulfur-hexafluoride [SF6]) performed by an experienced surgeon (I.P.). The control group consisted of the fellow eye of each patient and one eye randomly selected from 89 healthy volunteers.

Inclusion criteria were as follows:

- RRD Group: Patients who had undergone successful PPV ± SB, with complete resorption of SF6 at the time of testing.
- Both Groups: Ability to perform the tests.

Exclusion criteria for both groups included any condition that could affect the BCVA or hinder fundus visualization, such as corneal leukoma, clinically significant cataract, age-related macular degeneration (AMD), diabetic retinopathy (DR), previous vascular obstruction, epiretinal membrane (ERM), macular hole, proliferative vitreoretinopathy (PVR) of any grade, subretinal fluid (SRF) or intraretinal fluid (IRF) of any etiology, neuropathy or amblyopia of the studied eye.

A detailed clinical history was obtained from all participants which included the following data: sex, age, studied eye (right eye [RE], left eye [LE]), personal medical history, BCVA measured with Snellen charts and converted to the minimal angle of resolution (LogMAR), axial length (AL) measured in mm with the Aladdin KR-1W Series optical biometry system (Topcon Corporation, Tokyo, Japan), and intraocular pressure (IOP) measured with Goldmann applanation tonometry (mmHg). For the RRD group, additional data were collected: the duration of symptoms before seeking medical attention (days), time from symptom onset to surgery (days), and time from surgery to testing (days). Macular status prior to the surgery was also assessed using swept-source OCT (SS-OCT) Deep Range Imaging (DRI)-Triton SS-OCT (Topcon Corporation, Tokyo, Japan).

Macular retinal sensitivity was assessed using MAIA MP (Macular Integrity Assessment system, CenterVue, Padova, Italy), which projects stimuli of a predetermined intensity at specific points on the retina. In this study, we used the “Expert Exam” protocol, which follows a 4–2 strategy. This strategy begins with higher intensity stimuli and progressively decreases the intensity until the patient can no longer detect them. The macular integrity index and average total threshold were recorded in decibels (dB).

The studied area was defined by the circular grid provided by the MAIA system, covering an area with a 1500-micron radius centered on the fovea, where 37 points were stimulated. The grid was divided into three concentric rings: central (C), inner (I), and outer (O). Each inner ring was further subdivided into four quadrants: superior (S), temporal (T), inferior (I), and nasal (N). Three stimuli were projected within each quadrant, and the average sensitivity for each quadrant was used for analysis. The central point was analyzed individually and collectively with the 12 surrounding points (within the C circle), a measure referred to as “C global”.

**Figure 1:**
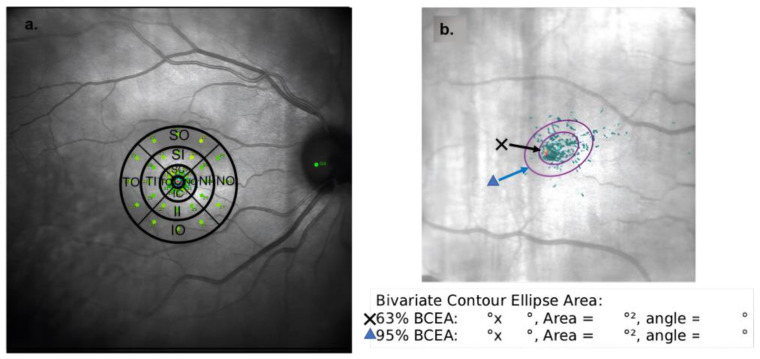
(**a**). Macular Integrity Assessment (MAIA) microperimetry sensitivity map with a superimposed grid showing the division into rings and quadrants. C, central; SC, superior central; TC, temporal central; IC, inferior central; NC, nasal central; SI, superior inner; TI, temporal inner; II, inferior inner; NI, nasal inner; SO, superior outer; TO, temporal outer; IO, inferior outer; NO, nasal outer. (**b**). Fixation plot. The eye tracker records fixation points which are grouped into two areas representing 63% and 95% of the points, known as the bivariate contour ellipse areas (BCEA). The device provides a quantitative analysis of each BCEA, including the area and angle that defines their orientation.

The MAIA system includes an integrated “eye tracker” that analyzes fixation using different parameters: fixation stability (P1 and P2 in %), fixation losses (%), and bivariate contour ellipse (BCEA). The BCEA quantifies the dispersion of eye movements during the test, generating ellipses encompassing 63% and 95% of the fixation points. The dispersion of points in two perpendicular directions—horizontal and vertical—provides an angle (in degrees) for each ellipse, indicating its orientation (Figure 1).

**Figure 2:**
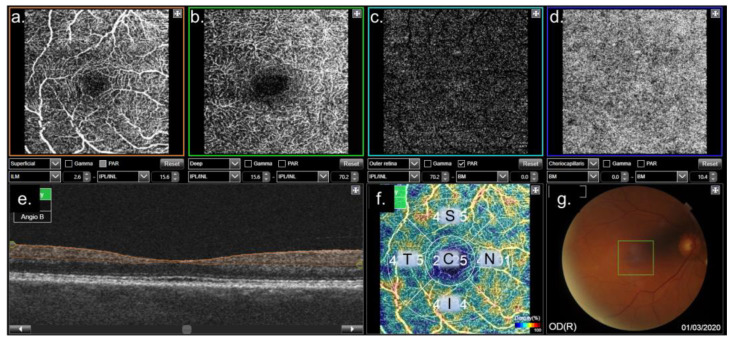
Example of the results obtained from a 3 × 3 OCTA using the DRI-Triton OCT device. (**a**) Retinal superficial capillary plexus (SCP). (**b**) Retinal deep capillary plexus (DCP). (**c**) Outer retina. (**d**) Choriocapillaris (CC). (**e**) OCT profile with the area of vessel density (VD) analysis highlighted in orange (in this example, the SCP is shown). (**f**) Vessel density in the analyzed area, represented as the percentage of pixels occupied by blood flow, divided into five sectors: S, superior; T, temporal; I, inferior; N, nasal; C, central. (**g**) Retinography of the posterior pole, showing the examined area marked as a green square.

After pupil dilation with mydriatic drops (Tropicamide^®^; Alcon Cusí, Barcelona, Spain), OCTA was performed using the DRI-Triton SS-OCT (Topcon Corporation, Tokyo, Japan). A 6 × 6 mm macular three-dimensional scan was obtained, along with a 3 × 3 mm OCTA scan, using IMAGEnet 6 software (Version 1.22.1.14101©; Topcon Corporation, Tokyo, Japan). The device provided vessel density (VD) measurements for the SCP, DCP, and CC. It divides the circular macular area into five sectors: a central circular sector (C) and four concentric sectors, superior (S), temporal (T), inferior (I), and nasal (N). The minimum required image resolution was set at 65 out of 100 (Figure 2).

The foveal avascular zone (FAZ) area, along with its vertical (Ver. D) and horizontal (Hor. D) diameters in the SCP and DCP, were manually delineated three times by two independent ophthalmologists (I.B., I.P.) using the measurement tool provided by the imaging system. The final reported values are expressed as the mean ± standard deviation (SD) of these measurements.

A list of abbreviations used is provided in the Supplementary Materials.

### Statistical Analysis

Data collection and statistical analysis were performed using SPSS software (SPSS 25, SPSS Inc., IBM Corporation, Armonk, NY, USA). Normal data distribution was assessed using the Kolmogorov–Smirnov test. Given that most parameters did not conform to a normal distribution, non-parametric tests were employed for statistical analysis. Differences between groups were identified with the Mann–Whitney U test for independent samples, while relationships between variables were analyzed using Spearman’s correlation coefficient. A *p*-value of <0.05 was considered statistically significant.

## 3. Results

### 3.1. Descriptive and Clinical Data

The initial RRD group included 65 eyes. However, 18 patients were excluded for the following reasons: 7 were unable to complete the tests, 4 had SRF, 5 presented ERM, 1 had subfoveal outer retinal layer disruption, and 1 had cystoid macular edema (CME). In these cases, both the RRD-affected eye and the fellow eye were excluded from the analysis. Consequently, 47 eyes from the RRD group were included in the final analysis. The control group consisted of 136 eyes. Demographic and clinical data for both groups are presented in Table 1.

There were no statistically significant differences between groups in terms of age, sex, laterality of the studied eye, or IOP. However, there were significant differences in BCVA at the time of the examination with the RRD group having worse BCVA compared to the control group (*p* < 0.001). Statistically significant differences were noted in the AL, which was significantly larger in the RRD group (*p* < 0.001).

Regarding macular function, statistically significant differences were observed in all sectors assessed by MAIA (*p* < 0.001, *p* = 0.002 in SO), macular integrity, and average threshold. The control group exhibited better macular sensitivity overall. However, no significant differences were found in any of the fixation parameters.

Table 2 presents the OCTA data collected for both groups. Statistically significant differences were observed in the SCP for the S (*p* = 0.024) and N (*p* = 0.016) sectors, as well as in the Ver. D of the FAZ (*p* = 0.008). In the DCP, significant differences were observed in the S (*p* = 0.048) and C (*p* = 0.015) sectors, as well as in the area (*p* = 0.026) and Ver. D of the FAZ (*p* = 0.001). No significant differences were found in the CC.

### 3.2. Correlations Within the Control Group

Correlations between OCTA, BCVA, and MAIA MP within the control group are presented as Supplementary Table S1. Values that reached statistically significance levels are presented in Table 3.

The FAZ was the OCTA parameter showing the strongest correlations, especially the Ver. D.

In the SCP, the Ver. D showed positive correlations with the following MAIA sectors: TO (r = 0.403), IO (r = 0.339), NO (r = 0.275), SI (r = 0.307), TI (r = 0.487), II (r = 0.367), NI (r = 0.400), SC (r = 0.406), IC (r = 0.340), NC (r = 0.319), and C global (r = 0.300). Additionally, Ver. D correlated positively with average threshold (r = 0.405) and fixation stability P1 (r = 0.360) while showing a negative correlation with macular integrity (r = −0.306) and the BCEA 63% (r = −0.338) and 95% areas (r = −0.313) of the MAIA.

In the DCP, the Ver. D was positively correlated with TO (r = 0.332), IO (r = 0.308), TI (r = 0.402), II (r = 0.375), NI (r = 0.378), SC (r = 0.344), TC (r = 0.269), IC (r = 0.303), NC (r = 0.289), and C global (r = 0.290) sectors, average threshold (r = 0.358), and fixation stability P1 (r = 0.419) and P2 (r = 0.392). Negative correlations were also noted with both BCEA 63% (r = −0.382) and 95% areas (r = −0.350) of the MAIA. The FAZ area in the DCP was correlated with the average threshold (r = 0.331) and most MAIA sectors, but not with the fixation parameters.

BCVA showed negative correlations with all MAIA sectors (*p* < 0.01) as detailed in Table 3. Additionally, BCVA correlated with macular integrity (r = 0.355, *p* < 0.001) and average threshold (r = −0.370, *p* < 0.001).

Correlations between OCTA and BCVA were primarily observed in relation to the FAZ. In the SCP, BCVA was correlated with the FAZ area (r = −0.268, *p* = 0.050), Ver. D (r = −0.462, *p* = <0.001) and Hor. D (r = −0.352, *p* = 0.009). In the DCP, correlations were found with the FAZ area (r = −0.304, *p* = 0.026), Ver. D (r = −0.402, *p* = 0.003), and Hor. D (r = −0.320, *p* = 0.018).

### 3.3. Correlations Within the RRD Group

Table 4 presents the significant correlations between OCTA, BCVA, and MAIA MP within the RRD group. The remaining data are provided in Supplementary Table S2.

BCVA showed significant negative correlations with several MAIA MP sectors, including the SE (r = −0.287), TO (r = −0.311), SI (r = −0.344), TI (r = −0.293), TC (r = −0.255), C global (r = −0.344) sectors, as well as with the average threshold (r = −0.323), P1 (r = −0.356), P2 (r = −0.313). In contrast, it demonstrated positive correlations with BCEA 63% (r = 0.367) and BCEA 95% (r = 0.367) areas of the MAIA.

No significant correlations were found between any OCTA parameter and BCVA in the RRD group.

The time from surgery to testing showed positive correlations with MAIA MP sectors, including the TI (r = 0.288, *p* = 0.022), C (r = 0.299, *p* = 0.017), TC (r = 0.297, *p* = 0. 018), IC (r = 0.290, *p* = 0.021), and C global (r = 0.302, *p* = 0.16) sectors. It showed negative correlations with fixation stability parameters P1 (r = −0.273, *p* = 0.032) and P2 (r = −0.268, *p* = 0.035), as well as with fixation losses (r = −0.388, *p* = 0.002). A negative correlation was also observed with the Hor. D of the FAZ in the DCP (r = −0.323, *p* = 0.031). No correlation was found with BCVA.

The duration of symptoms before seeking medical attention showed negatively correlated only with the IO sector of the MAIA MP (r = −0.299, *p* = 0.16). In OCTA parameters, it negatively correlated with the FAZ area (r = −0.354, *p* = 0.016) and Hor. D (r = −0.301, *p* = 0.042) of the DCP. Again, no correlation was observed with BCVA.

Lastly, the time from symptom onset to surgery did not show any significant correlations with MAIA, OCTA, or BCVA.

## 4. Discussion

Changes in the FAZ and VD following RRD have been extensively studied, although often in small and heterogeneous patient groups. The results vary widely, as do the correlations established with BCVA [6]. To our knowledge, most studies on the FAZ focus primarily on its area with few subdividing the vascular plexuses into sectors, as we have done in our study and also performed in the research conducted by Nassar et al. [11]. Separate analyses of each plexus could help clarify which retinal layers are more affected and thus potentially more implicated in visual function impairment. In our study, we observed a decrease in the Ver. D of the FAZ in both SCP and DCP, as well as a reduction in the FAZ area in DCP. These findings contrast with the majority of studies [12].

We hypothesize that the observed changes are due to FAZ remodeling following surgery for several reasons. First, retinal contraction could reduce the FAZ area. Additionally, the activation of angiogenic factors following ischemia caused by RRD may contribute to an increase in central VD [13]. These changes might develop progressively during an extended follow-up period, which may explain why they are not detected in many studies. However, research by Wang et al. [14] and Stoebener et al. [15] found that patients with RRD initially experienced a decrease in VD at 2 and 4 weeks post-surgery, respectively, with an increase observed at 12 weeks and 6 months. In our study, we found a negative correlation between the time from surgery to testing and the Hor. D of the DCP.

Second, the significant differences observed in the Ver. D of both the SCP and DCP between the RRD and control groups could be attributed to retinal displacement following PPV. This phenomenon, with an incidence ranging from 6.4% to 62.8% of cases, involves a shift in major retinal vessels from their original position, typically along the vertical axis [16].

Finally, the FAZ can vary significantly between individuals, although both eyes of the same individual tend to show high symmetry when examined under similar conditions [7,13,17]. Our control group included both the fellow eyes of patients with RRD and eyes from healthy volunteers, which likely helped account for inter-subject variability.

In the pursuit of OCTA biomarkers that may predict visual function after an RRD, most authors use BCVA as the primary visual outcome. These results have been inconsistent, with some studies reporting no correlation [18,19], while others show positive correlations between VD in different plexuses and BCVA [14,15,20,21], whereas several studies have reported negative correlations between the FAZ area and BCVA [22,23,24]. We did observe a statistically significant correlation between BCVA and the FAZ area and its diameters in the control group. However, no such correlation was found in the RRD group. Furthermore, in the control group, all MAIA MP sectors, macular integrity and average threshold, were correlated with BCVA. In the RRD group, this correlation persisted only in specific sectors (S, T and C) and the average threshold. Additionally, BCVA in the RRD group showed significant correlations with fixation stability (P1 and P2) and BCEA (63% and 95% area). This reinforces the recommendation that visual function assessments should extend beyond BCVA alone [25].

In healthy eyes, a properly functioning retina is supported by adequate microvascularization, which contributes to predictable BCVA outcomes. However, in the presence of retinal damage, this balance is disrupted. The time from surgery to testing did not correlate with BCVA but did show correlations with OCTA and MAIA parameters, suggesting that neuroadaptation in the visual pathway enables patients to achieve better visual acuity outcomes than expected [26,27]. Various MP devices have been used in conditions involving central vision loss, demonstrating promising results in improving visual performance through biofeedback training focused on fixation abilities [28]. Specifically, Sahli et al. [29] used the MAIA MP device to rehabilitate 35 patients with retinal pathologies, showing significant improvements in fixation stability (P1; *p* = 0.003) and 95% BCEA (*p* = 0.018) after 10 sessions of biofeedback training. Patients also improved their scores on the National Eye Institute Visual Function Questionnaire (NEI-VFQ-25) (*p* < 0.001). We believe that the absence of visual stimuli in certain retinal areas could interfere with functional improvement. This may explain why, in our research, despite positive correlations between several MAIA sectors (TI, C, TC, IC, C global) and time from surgery to testing, fixation stability (P1 and P2) and fixation losses worsened over time. These findings are in line with studies on visual rehabilitation in patients with occipital strokes where various techniques to improve visual function have been explored [30]. It has been demonstrated that early and intensive training can significantly influence visual prognosis. Based on this, we believe there are compelling reasons to propose that patients who have undergone RRD repair should be assessed with MP in the first postoperative weeks. This would allow for mapping of the affected areas and the immediate design of appropriate rehabilitation strategies. Although the specific approach is beyond the scope of this study, we consider it an exciting area for future research.

Our findings indicate that recovery is influenced by time. Specifically, the duration between surgery and the MAIA test positively correlated with multiple MAIA sectors, while fixation parameters showed negative correlations. This suggests that time plays a role in shaping functional recovery outcomes.

Interestingly, we observed a negative correlation between only one MAIA sector (IO) and the time from symptom onset to the first medical examination. This result may seem counterintuitive, as longer delays in seeking medical attention are generally associated with worse visual prognoses. However, this discrepancy could be attributed to other factors, such as the condition of the macula prior to surgery, which likely plays a more significant role in determining visual outcomes.

In our study, the absence of subdivisions based on macular status or the extent of retinal detachment, combined with strict inclusion criteria requiring good fixation capacity and a minimum BCVA for eligibility, may have influenced this variable. Future studies with a more detailed stratification of pre-surgical macular condition and detached retina percentage are needed to further explore these relationships.

To our knowledge, only two studies have examined the correlation between OCTA and retinal function as measured by MP in patients following RRD surgery. Zabel et al. [9] analyzed 20 eyes with macula-ON RRD managed by SB and compared them with their 20 corresponding fellow eyes. Despite finding no differences in BCVA between groups, regardless of the extent of the RRD, they observed decreased retinal sensitivity across all MAIA sectors and reduced VD in the SCP, DCP, and radial peripapillary capillaries (RPC). However, only the VD of both SCP and RPC showed a statistically significant positive correlation with the average threshold. While the decline in retinal sensitivity mirrors our findings, we observed differences in BCVA, which could be attributed to our inclusion of both macula-ON and macula-OFF RRD cases, as well as the sample size.

Nassar et al. [11] conducted a study evaluating microvascular and functional changes using MP with the OPTOS Spectral OCT/SLO (scanning laser ophthalmoscope) (OPTOS, Inc., FL, USA) in 30 patients who had undergone RRD surgery before and after silicone oil removal. Despite they did not find statistically significant correlations at the macular level, they observed statistically significant correlations between MP and CC at the optic nerve level (*p* = 0.031). However, given the distinct characteristics and specific indications of silicone oil as a tamponade, we believe the patient population in their study differs substantially from ours, making direct comparisons challenging.

To our knowledge, no previous studies have explored correlations between CC and MAIA parameters. In our study, we identified sparse correlations in both groups. Specifically, in the control group, we observed positive correlations between the C sector of the CC and most MAIA sectors, a pattern that contrasts with the values observed in the SCP and DCP. These findings suggest potential differences in functional significance between vascular layers. However, further investigation is necessary to clarify the functional relevance of these observations.

The limitations of our study include its cross-sectional design, which prevents the evaluation of longitudinal changes in patients with RRD. Future analyses would benefit from a longitudinal approach, categorizing patients based on their initial macular status and the extent of RRD prior to surgery. Additionally, a larger cohort would enhance the robustness of the findings. Another limitation is that our control group partially consisted of contralateral eyes. While this approach helps mitigate inter-individual variability in factors such as the FAZ, it may not fully account for other variations that could influence the outcomes.

As far as we are aware, our study is the first to establish correlations between OCTA, MAIA, and BCVA in a control group. Furthermore, it is the first to analyze sector-specific data from both OCTA and MAIA in both healthy and those eyes with RRD, and to assess fixation parameters in this context.

We believe that emerging technologies, which allow for the evaluation of retinal function and microvascularization, should be integrated into the routine assessment of anatomical changes and visual performance in patients post-RRD surgery. Additionally, MP should be considered as a key tool in the visual evaluation and rehabilitation of these patients, offering valuable insights into their recovery and functional outcomes.

## Figures and Tables

**Table 1:** Demographic and clinical data of both groups. RRD, rhegmatogenous retinal detachment; BCVA, best corrected visual acuity; AL, axial length; IOP, intraocular pressure; SD, standard deviation. Statistically significant differences are presented in bold and shaded in gray. In this case, all parameters reached *p* < 0.0001.

		RRD Group	Control Group
**Examined eye**	Total (*n*, %)	*n* = 47	*n* = 136
	Right eye	20 (42.6%)	73 (53.7%)
	Left eye	27 (57.5%)	63 (46.3%)
**Sex (*n*, %)**	Female	17 (36.2%)	63 (46.3%)
	Male	30 (63.8%)	73 (53.7%)
**Age (years ± SD)** (range)		58.81 ± 9.31 (33.00–74.00)	59.08 ± 10.02 (33.00–84.00)
**BCVA** **(LogMAR scale ± SD)** (range)		**0.026 ± 0.29** **(0.00–1.00)**	**0.063 ± 0.12** **(0.00–1.00)**
**AL (mm ± SD)** (range)		**25.72 ± 2.93** **(21.61–24.20)**	**24.49 ± 2.79** **(21.51–24.87)**
**IOP (mmHg ± SD)** (range)		14.35 ± 2.67 (9–21)	13.49 ± 2.65 (8–20)
**Duration of symptoms** **(days ± SD)** (range)		4.66 ± 5.95 (0–30)	
**Time between onset the symptoms and surgery** **(days ± SD)** (range)		9.20 ± 6.40 (1–35)	
**Time between surgery to tests (days ± SD)** (range)	Less than 1 year (*n* = 24)	129.13 ± 122.86 (30–356)	
	Between 1 and 4 years (*n* = 23)	682.69 ± 427.90 (369–1458)	
	Total	474.90 ± 620.36 (30–1458)	

**Table 2:** Differences in OCTA measurements between the RRD group and the control group. The result is expressed as mean ± SD. Abbreviations: SD, standard deviation; FAZ, foveal avascular zone; SCP, superficial capillary plexus; DCP, deep capillary plexus; VD, vessel density; Ver. D, vertical diameter; Hor. D, horizontal diameter; S, superior; T, temporal; I, inferior; N, nasal; C, central. Statistically significant differences (*p* < 0.05) are presented in bold and shaded in gray.

			RRD Group	Control Group
**SCP**	VD	S	**47.12 ± 4.27**	**49.04 ± 4.15**
		T	46.26 ± 3.75	46.88 ± 3.00
		I	45.66 ± 3.20	46.45 ± 3.29
		N	**48.52 ± 4.92**	**50.24 ± 4.39**
		C	22.03 ± 4.74	20.77 ± 4.32
	FAZ	Area	233.30 ± 156.89	251.07 ± 90.72
		Ver. D	**434.48 ± 174.89**	**505.33 ± 142.83**
		Hor. D	439.45 ± 190.47	491.06 ± 138.89
**DCP**	VD	S	**49.69 ± 4.32**	**50.93 ± 4.66**
		T	47.34 ± 6.08	47.30 ± 3.44
		I	48.69 ± 3.61	49.33 ± 3.69
		N	51.95 ± 4.41	52.55 ± 4.46
		C	**24.85 ± 6.39**	**21.48 ± 5.97**
	FAZ	Area	**189.90 ± 104.27**	**236.40 ± 102.61**
		Ver. D	**379.81 ± 159.29**	**503.81 ± 169.85**
		Hor. D	449.11 ± 180.31	504.50 ± 126.35
**CC**	VD	S	51.26 ± 2.70	51.99 ± 2.91
		T	53.16 ± 2.46	53.33 ± 2.45
		I	52.67 ± 2.40	52.59 ± 2.83
		N	52.68 ± 2.42	52.97 ± 3.00
		C	48.77 ± 4.31	49.06 ± 3.68

**Table 3:** Correlations between OCTA sectors, BCVA, and MAIA data within the control group (this table only shows the data concerning those correlations that have reached statistical significance. The complete table can be found in Supplementary Material Table S1). FAZ, foveal avascular zone; SCP, superficial capillary plexus; DCP, deep capillary plexus; Ver. D, vertical diameter; Hor. D, horizontal diameter; S, superior; T, temporal; I, inferior; N, nasal; C, central; BCVA, best corrected visual acuity; MP, microperimetry; SO, superior outer; TO, temporal outer; IO, inferior outer; NO, nasal outer; SI, superior inner; TI, temporal inner; II, inferior inner; NI, nasal inner; C, central point; SC, superior central; TC, temporal central; IC, inferior central; NC nasal central; C global, central global point; BCEA, bivariate contour ellipse; Cc, Statistical correlation coefficient; Sig., Statistical significance. Statistically significant correlations at *p* < 0.05 are highlighted in light gray, and those at *p* < 0.01 are highlighted in dark gray.

Control Group				MP SECTORS																Fixation Stability		BCEA				
				SO	TO	IO	NO	SI	TI	II	NI	C	SC	TC	IC	NC	C Global	Macular Integrity	Average Threshold	P1	P2	63 Area	63 Angle	95 Area	95 Angle	Fixation Losses
SCP	T	Cc												−0.310												
		Sig.												0.023												
	I	Cc													−0.293	−0.318	−0.269									
		Sig.													0.031	0.019	0.049									
	N	Cc			−0.348			−0.290	−0.311	−0.296		−0.310		−0.368	−0.299		−0.271		−0.279							−0.335
		Sig.			0.010			0.033	0.022	0.029		0.023		0.006	0.028		0.047		0.041							0.013
	FAZ	Area	Cc						0.358	0.269			0.291						0.302							
			Sig.						0.008	0.049			0.032						0.027							
		Ver. D	Cc		0.403	0.339	0.275	0.307	0.487	0.367	0.400		0.406		0.340	0.319	0.300	−0.306	0.405	0.360	0.347	−0.338		−0.313		
			Sig		0.003	0.012	0.044	0.024	0.000	0.006	0.003		0.002		0.012	0.019	0.027	0.024	0.002	0.008	0.011	0.012		0.021		
		Hor. D	Cc						−0.309				0.270													
			Sig.						0.023				0.048													
DCP	T	Cc			−0.284																					
		Sig.			0.037																					
	N	Cc												−0.274												
		Sig.												0.045												
	C	Cc					−0.280		−0.323				−0.335													
		Sig.					0.040		0.017				0.013													
	FAZ	Area	Cc		0.329	0.312	0.288		0.455	0.319	0.310		0.347	0.326	0.341	0.285	0.285		0.331							
			Sig.		0.015	0.022	0.034		0.001	0.019	0.023		0.010	0.016	0.012	0.037	0.037		0.015							
		Ver. D	Cc		0.332	0.308			0.412	0.375	0.378		0.344	0.269	0.303	0.289	0.290		0.358	0.419	0.392	−0.382		−0.350		
			Sig		0.014	0.024			0.002	0.005	0.005		0.011	0.049	0.026	0.034	0.033		0.008	0.002	0.004	0.004		0.009		
		Hor. D	Cc			0.281														0.312	0.276	−0.324		−0.302		
			Sig.			0.040														0.023	0.046	0.017		0.026		
CC	S	Cc																		−0.388	−0.367	0.362		0.349		
		Sig.																		0.004	0.007	0.007		0.010		
	T	Cc																			0.275			−0.272		
		Sig.																			0.047			0.047		
	I	Cc																				−0.275				
		Sig.																				0.044				
	C	Cc			0.296	0.298				0.315	0.328	0.304	0.273	0.268	0.339		0.282		0.315							
		Sig.			0.030	0.029				0.020	0.015	0.025	0.046	0.050	0.012		0.039		0.020							
BCVA		Cc		−0.288	−0.260	−0.243	−0.291	−0.280	−0.345	−0.308	−0.281	−0.261	−0.309	−0.279	−0.330	−0.289	−0.299	0.355	−0.370							
		Sig		0.001	0.002	0.004	0.001	0.001	< 0.001	< 0.001	0.001	0.002	< 0.001	0.001	< 0.001	0.001	< 0.001	< 0.001	< 0.001							

**Table 4:** Correlations between OCTA sectors, BCVA, and MAIA data within the RRD group (this table only shows the data concerning those correlations that have reached statistical significance. The complete table can be found in Supplementary Material Table S2). FAZ, foveal avascular zone; SCP, superficial capillary plexus; DCP, deep capillary plexus; VD, vessel density; Ver. D, vertical diameter; S, superior; T, temporal; I, inferior; N, nasal; C, central; BCVA, best corrected visual acuity; MP, microperimetry; SO, superior outer; TO, temporal outer; IO, inferior outer; NO, nasal outer; SI, superior inner; TI, temporal inner; II, inferior inner; NI, nasal inner; C, central point; SC, superior central; TC, temporal central; IC, inferior central; NC nasal central; C global, central global point; BCEA, bivariate contour ellipse; Cc, Statistical correlation coefficient; Sig., Statistical significance. Statistically significant correlations at *p* < 0.05 are highlighted in light gray, and those at *p* < 0.01 are highlighted in dark gray.

RRD Group				MP SECTORS																Fixation Stability		BCEA				
				SO	TO	IO	NO	SI	TI	II	NI	C	SC	TC	IC	NC	C Global	Macular Integrity	Average Threshold	P1	P2	63 Area	63 Angle	95 Area	95 Angle	Fixation Losses
SCP	S	Cc										0.314	0.339													
		Sig.										0.032	0.020													
	I	Cc				−0.298				−0.376																
		Sig.				0.042				0.009																
	N	Cc																					0.306			
		Sig.																					0.036			
	C	Cc																					−0.293			
		Sig.																					0.046			
DCP	C	Cc							0.297														−0.464		−0.482	
		Sig.							0.043														0.001		0.001	
	FAZ	Area	Cc							−0.289															0.290	
			Sig.							0.049															0.048	
		Ver. D	Cc																				0.345		0.341	
			Sig																				0.017		0.019	
CC	S	Cc										0.409	0.333									0.290		0.292		
		Sig.										0.004	0.022									0.048		0.047		
	T	Cc																					0.325		0.301	
		Sig.																					0.026		0.040	
BCVA		Cc		−0.287	−0.311			−0.344	−0.293					−0.324		−0.255	−0.344		−0.323	−0.356	−0.313	0.367		0.367		
		Sig.		0.021	0.012			0.005	0.18					0.008		0.041	0.005		0.009	0.004	0.012	0.003		0.003		

## Data Availability

The data presented in this study are available on request from the corresponding author. The data are not publicly available due to privacy and ethical restrictions.

## Supplementary Materials

The following supporting information can be downloaded at: https://www.mdpi.com/article/10.3390/biomedicines12122911/s1, Table S1. Correlations between OCTA sectors, BCVA, and MAIA data within the control group. Table S2. Correlations between OCTA sectors, BCVA, and MAIA data within the RRD group.

## Abbreviations

The following abbreviations are used in this manuscript:

AL	axial lenght
AMD	age-related macular degeneration
BCEA	bivariate contour ellipse
BCVA	best corrected visual acuity
C	central
CC	choriocapillaris
Cc	correlation coefficient
CME	cystoid macular edema
dB	decibels
DCP	deep capillary plexus
DM	diabetes mellitus
DR	diabetic retinopathy
DRI	deep range imaging
ERM	epiretinal membrane
FAZ	foveal avascular zone
Hor. D	horizontal diameter
I	(being the second letter of the abbreviation) inner
I	(being the first letter of the abbreviation) inferior
IC	inferior central
ICP	intermediate capillary plexus
II	inferior inner
IO	inferior outer
IOP	intraocular pressure
IRF	intraretinal fluid
LE	left eye
MAIA	macular integrity assessment
MP	microperimetry
N	nasal
n	sample size
NC	nasal central
NI	nasal inner
NO	nasal outer
O	outer
OCTA	optical coherence tomography angiography
PPV	pars plana vitrectomy
PVR	proliferative vitreoretinopathy
RE	right eye
RRD	rhegmatogenous retinal detachment
S	superior
SB	scleral buckling
SC	superior central
SCP	superficial capillary plexus
SD	standard deviation
SF6	sulfur-hexafluoride
SI	superior inner
Sig	statistical significance
SO	superior outer
SRF	subretinal fluid
SS-OCT	swept-source optical coherence tomography
T	temporal
TC	temporal central
TI	temporal inner
TO	temporal outer
Ver. D	vertical diameter
VD	vessel den
