# Supplementary material for: Impact of Rhegmatogenous Retinal Detachment on Macular Vascular and Functional Integrity
Source: Biomedicines. 2024 Dec 20;12(12):2911. doi: 10.3390/biomedicines12122911 (PMC11673870; doi:10.3390/biomedicines12122911)
Supplement: Supplementary file 1 [file biomedicines-12-02911-s001.zip › biomedicines-3360428-supplementary.pdf]

Supplementary Material

Table S1. Correlations between OCTA sectors, BCVA, and MAIA data within the control group. FAZ, foveal avascular zone; SCP, superficial capillary plexus; DCP, deep capillary plexus; VD, vessel density; Ver. D, vertical diameter; Hor. D, horizontal diameter; S, superior; T, temporal; I, inferior; N, nasal; C, central; BCVA, best corrected visual acuity; MP, microperimetry; SO, superior outer; TO, temporal outer; IO, inferior outer; NO, nasal outer; SI, superior inner; TI, temporal inner; II, inferior inner; NI, nasal inner; C, central point; SC, superior central; TC, temporal central; IC, inferior central; NC nasal central; C global, central global point; BCEA, bivariate contour ellipse. Statistically significant correlations at p<0.05 are highlighted in light grey, and those at p<0.01 are highlighted in dark grey.

Control group			MP SECTORS																Fixation stability		BCEA				
			SO	TO	IO	NO	SI	TI	II	NI	C	SC	TC	IC	NC	C global	Macular integrity	Average threshold	P1	P2	63 area	63 angle	95 area	95 angle	Fixation losses
SCP	S	Cc	-0.073	-0.218	-0.079	-0.042	-0.064	-0.208	-0.184	0.003	-0.108	-0.108	-0.107	-0.060	0.006	-0.083	0.062	-0.071	-0.069	-0.067	0.049	0.051	0.047	0.060	-0.101
		Sig.	0.599	0.144	0.570	0.765	0.644	0.131	0.182	0.982	0.438	0.439	0.443	0.666	0.957	0.553	0.657	0.612	0.621	0.631	0.723	0.716	0.734	0.671	0.467
	T	Cc	-0.211	-0.262	-0.179	-0.200	-0.066	-0.235	-0.237	-0.211	-0.009	-0.264	-0.310	-0.243	-0.166	-0.240	0.154	-0.213	0.006	0.055	-0.014	-0.092	-0.018	-0.089	-0.121
		Sig.	0.125	0.056	0.195	0.148	0.624	0.087	0.084	0.126	0.950	0.054	0.023	0.077	0.231	0.080	0.266	0.121	0.963	0.695	0.923	0.511	0.898	0.527	0.382
	I	Cc	-0.187	-0.220	-0.167	-0.225	-0.194	-0.206	-0.130	-0.204	0.013	-0.247	-0.266	-0.293	-0.318	-0.269	0.263	-0.206	0.066	0.110	-0.097	0.000	-0.074	0.013	-0.111
		Sig.	0.177	0.109	0.228	0.102	0.160	0.135	0.349	0.140	0.924	0.072	0.052	0.031	0.019	0.049	0.055	0.132	0.637	0.432	0.486	1.000	0.594	0.924	0.426
	N	Cc	-0.252	-0.348	-0.300	-0.223	-0.290	-0.311	-0.296	-0.265	-0.310	-0.210	-0.368	-0.299	-0.243	-0.271	0.203	-0.279	-0.099	-0.066	0.112	-0.116	0.111	-0.110	-0.335
		Sig.	0.067	0.010	0.027	0.105	0.033	0.022	0.029	0.053	0.023	0.128	0.006	0.028	0.076	0.047	0.142	0.041	0.480	0.641	0.419	0.408	0.432	0.432	0.013
	C	Cc	0.004	-0.080	-0.009	-0.055	-0.032	-0.194	-0.051	-0.069	-0.017	-0.137	-0.074	-0.044	-0.044	-0.057	0.051	-0.091	-0.172	-0.248	0.215	0.172	0.219	0.178	0.061
		Sig.	0.557	0.566	0.949	0.693	0.816	0.160	0.714	0.620	0.904	0.326	0.596	0.752	0.752	0.683	0.715	0.512	0.217	0.073	0.119	0.219	0.111	0.202	0.660
FAZ	Area	Cc	0.156	0.211	0.228	0.184	0.168	0.358	0.269	0.256	0.183	0.291	0.246	0.260	0.227	0.214	-0.234	0.302	0.161	0.181	-0.161	-0.214	-0.145	-0.206	0.150
		Sig.	0.261	0.126	0.097	0.184	0.223	0.008	0.049	0.061	0.185	0.032	0.073	0.058	0.099	0.119	0.088	0.027	0.249	0.194	0.243	0.123	0.294	0.138	0.279
	Ver. D	Cc	0.229	0.403	0.339	0.275	0.307	0.487	0.367	0.400	0.176	0.406	0.266	0.340	0.319	0.300	-0.306	0.405	0.360	0.347	-0.338	-0.142	-0.313	-0.130	0.262
		Sig	0.097	0.003	0.012	0.044	0.024	0.000	0.006	0.003	0.204	0.002	0.052	0.012	0.019	0.027	0.024	0.002	0.008	0.011	0.012	0.311	0.021	0.355	0.056
	Hor. D	Cc	0.039	0.218	0.176	0.157	0.240	-0.309	0.182	0.228	0.057	0.270	0.134	0.192	0.183	0.158	-0.200	0.259	0.213	0.209	-0.237	-0.023	-0.223	-0.019	0.155

		Sig.	0.777	0.113	0.203	0.258	0.081	0.023	0.189	0.097	0.682	0.048	0.332	0.164	0.185	0.254	0.148	0.059	0.125	0.133	0.085	0.873	0.106	0.892	0.264
DCP	S	Cc	-0.098	-0.109	0.004	0.067	-0.025	-0.163	-0.129	0.088	0.123	-0.124	-0.140	-0.055	-0.040	-0.062	0.011	-0.086	-0.107	-0.115	0.121	0.144	0.101	0.138	-0.219
		Sig.	0.480	0.432	0.978	0.630	0.856	0.238	0.353	0.528	0.377	0.370	0.312	0.695	0.775	0.657	0.935	0.536	0.446	0.411	0.385	0.305	0.465	0.325	0.112
	T	Cc	-0.238	-0.284	-0.204	-0.184	-0.123	-0.174	-0.144	-0.147	0.066	-0.238	-0.212	-0.179	-0.140	-0.160	0.229	-0.214	0.012	0.115	-0.013	-0.129	-0.046	-0.144	-0.090
		Sig.	0.083	0.037	0.139	0.183	0.374	0.209	0.298	0.289	0.635	0.064	0.123	0.195	0.311	0.247	0.096	0.120	0.929	0.410	0.927	0.357	0.739	0.302	0.518
	I	Cc	0.034	0.075	0.101	0.023	0.029	0.027	0.078	0.090	0.124	-0.027	-0.046	-0.026	-0.104	-0.032	0.153	0.018	0.070	0.035	-0.063	0.126	-0.048	0.130	0.000
		Sig.	0.810	0.588	0.469	0.866	0.834	0.848	0.577	0.519	0.373	0.846	0.740	0.851	0.456	0.816	0.269	0.897	0.618	0.801	0.650	0.368	0.732	0.353	0.998
	N	Cc	-0.167	-0.220	-0.065	-0.082	-0.133	-0.070	-0.075	-0.106	-0.083	-0.150	-0.274	-0.176	-0.143	-0.142	0.039	-0.135	-0.169	-0.086	0.169	-0.136	0.158	-0.142	-0.147
		Sig.	0.227	0.111	0.642	0.556	0.339	0.615	0.589	0.445	0.551	0.278	0.045	0.204	0.303	0.305	0.781	0.330	0.225	0.541	0.221	0.332	0.254	0.311	0.290
	C	Cc	-0.217	-0.188	-0.234	-0.280	-0.167	-0.323	-0.206	-0.221	-0.153	-0.335	-0.233	-0.191	-0.253	0.235	0.251	-0.259	-0.016	-0.089	0.069	0.232	0.062	0.224	-0.106
		Sig.	0.115	0.174	0.089	0.040	0.229	0.017	0.135	0.109	0.271	0.013	0.090	0.166	0.067	0.087	0.067	0.059	0.912	0.525	0.619	0.095	0.657	0.108	0.446
FAZ	Area	Cc	0.215	0.329	0.312	0.288	0.231	0.455	0.319	0.310	0.144	0.347	0.326	0.341	0.285	0.285	-0.249	0.331	0.203	0.188	-0.212	-0.131	-0.188	-0.124	0.175
		Sig.	0.119	0.015	0.022	0.034	0.092	0.001	0.019	0.023	0.297	0.010	0.016	0.012	0.037	0.037	0.070	0.015	0.145	0.177	0.124	0.350	0.173	0.377	0.206
	Ver. D	Cc	0.173	0.332	0.308	0.235	0.235	0.412	0.375	0.378	0.092	0.344	0.269	0.303	0.289	0.290	-0.250	0.358	0.419	0.392	-0.382	-0.095	-0.350	-0.066	-0.187
		Sig	0.212	0.014	0.024	0.088	0.088	0.002	0.005	0.005	0.507	0.011	0.049	0.026	0.034	0.033	0.068	0.008	0.002	0.004	0.004	0.496	0.009	0.542	0.176
	Hor. D	Cc	0.030	0.236	0.281	0.163	0.217	0.249	0.197	0.246	0.099	0.266	0.182	0.222	0.185	0.167	-0.162	0.251	0.312	0.276	-0.324	0.090	-0.302	0.098	0.156
		Sig.	0.828	0.086	0.040	0.238	0.115	0.070	0.154	0.073	0.477	0.052	0.188	0.106	0.181	0.228	0.241	0.067	0.023	0.046	0.017	0.519	0.026	0.485	0.260
CC	S	Cc	0.078	-0.166	-0.056	0.062	-0.105	-0.137	-0.040	-0.018	0.122	-0.073	-0.085	0.029	0.035	-0.057	-0.066	-0.027	-0.388	-0.367	0.362	-0.110	0.349	-0.103	0.066
		Sig.	0.573	0.224	0.690	0.657	0.451	0.322	0.774	0.900	0.378	0.598	0.542	0.834	0.804	0.684	0.635	0.848	0.004	0.007	0.007	0.433	0.010	0.463	0.627
	T	Cc	0.063	0.182	-0.048	0.073	0.109	0.064	-0.075	0.025	0.088	0.056	0.036	0.141	0.165	0.085	-0.108	0.054	0.237	0.275	-0.261	-0.090	-0.272	-0.091	0.092
		Sig.	0.653	0.189	0.732	0.599	0.433	0.645	0.592	0.859	0.525	0.687	0.795	0.309	0.234	0.540	0.437	0.699	0.088	0.047	0.056	0.520	0.047	0.519	0.508
	I	Cc	-0.038	0.059	-0.071	-0.058	0.061	-0.060	-0.129	-0.046	0.107	0.019	-0.104	-0.100	-0.106	-0.084	0.183	-0.077	0.203	0.230	-0.275	0.018	-0.267	0.022	0.075
		Sig.	0.784	0.669	0.612	0.675	0.663	0.667	0.351	0.741	0.440	0.894	0.452	0.471	0.444	0.545	0.185	0.579	0.145	0.098	0.044	0.898	0.051	0.876	0.591
	N	Cc	0.127	0.003	-0.036	0.104	0.005	0.065	0.047	-0.006	0.061	0.024	-0.007	0.004	0.097	0.053	-0.038	-0.007	-0.159	-0.098	0.124	-0.177	0.116	-0.177	-0.064
		Sig.	0.359	0.985	0.798	0.455	0.969	0.641	0.735	0.966	0.659	0.861	0.959	0.980	0.484	0.706	0.787	0.960	0.255	0.485	0.372	0.204	0.405	0.205	0.643
	C	Cc	0.257	0.296	0.298	0.157	0.243	0.262	0.315	0.328	0.304	0.273	0.268	0.339	0.208	0.282	-0.211	0.315	0.020	0.045	0.002	-0.063	0.005	-0.058	0.124
		Sig.	0.061	0.030	0.029	0.257	0.077	0.056	0.020	0.015	0.025	0.046	0.050	0.012	0.130	0.039	0.126	0.020	0.885	0.748	0.988	0.654	0.970	0.678	0.373
BCVA		Cc	-0.288	-0.260	-0.243	-0.291	-0.280	-0.345	-0.308	-0.281	-0.261	-0.309	-0.279	-0.330	-0.289	-0.299	0.355	-0.370	-0.151	-0.80	0.154	-0.021	0.144	-0.024	-0.035
		Sig	0.001	0.002	0.004	0.001	0.001	< 0.001	< 0.001	0.001	0.002	< 0.001	0.001	< 0.001	0.001	< 0.001	< 0.001	< 0.001	0.81	0.356	0.074	0.811	0.094	0.783	0.686

Table S2. Correlations between OCTA sectors, BCVA, and MAIA data within the RRD group. FAZ, foveal avascular zone; SCP, superficial capillary plexus; DCP, deep capillary plexus; VD, vessel density; Ver. D, vertical diameter; Hor. D, horizontal diameter; S, superior; T, temporal; I, inferior; N, nasal; C, central; BCVA, best corrected visual acuity; MP, microperimetry; SO, superior outer; TO, temporal outer; IO, inferior outer; NO, nasal outer; SI, superior inner; TI, temporal inner; II, inferior inner; NI, nasal inner; C, central point; SC, superior central; TC, temporal central; IC, inferior central; NC nasal central; C global, central global point; BCEA, bivariate contour ellipse. Statistically significant correlations at p<0.05 are highlighted in light grey, and those at p<0.01 are highlighted in dark grey.

RRD Group				MP SECTORS																Fixation stability		BCEA				
				SO	TO	IO	NO	SI	TI	II	NI	C	SC	TC	IC	NC	C global	Macular integrity	Average threshold	P1	P2	63 area	63 angle	95 area	95 angle	Fixation losses
SCP	S		Cc	0.158	0.090	-0.224	-0.200	0.223	0.151	-0.176	-0.035	0.314	0.339	0.078	0.107	0.154	0.242	0.088	0.006	0.115	0.165	-0.075	-0.047	-0.066	-0.024	0.065
			Sig.	0.288	0.547	0.129	0.178	0.132	0.312	0.237	0.816	0.032	0.020	0.603	0.475	0.303	0.101	0.556	0.970	0.446	0.273	0.617	0.753	0.659	0.872	0.663
	T		Cc	0.115	-0.066	-0.071	-0.015	0.065	0.067	-0.090	0.086	0.017	0.209	0.194	0.157	0.001	0.239	-0.102	0.129	0.101	0.025	-0.133	0.150	-0.128	0.155	-0.034
			Sig.	0.443	0.661	0.635	0.919	0.663	0.656	0.550	0.567	0.911	0.160	0.191	0.293	0.993	0.105	0.494	0.387	0.503	0.868	0.373	0.313	0.392	0.299	0.822
	I		Cc	-0.007	-0.179	-0.298	-0.117	-0.180	-0.221	-0.376	-0.028	0.114	-0.114	-0.044	-0.229	-0.169	-0.026	-0.013	-0.043	-0.145	-0.165	0.120	0.122	0.117	0.113	-0.010
			Sig.	0.961	0.229	0.042	0.434	0.225	0.135	0.009	0.854	0.447	0.447	0.771	0.122	0.257	0.862	0.932	0.773	0.337	0.273	0.422	0.414	0.434	0.449	0.945
	N		Cc	-0.094	-0.105	-0.194	-0.194	-0.043	-0.128	-0.242	-0.079	0.036	0.141	-0.145	-0.115	-0.035	0.004	-0.105	0.018	-0.158	-0.162	0.146	0.306	0.138	0.255	-0.189
			Sig.	0.528	0.482	0.192	0.192	0.777	0.391	0.101	0.599	0.810	0.346	0.331	0.442	0.813	0.978	0.484	0.906	0.294	0.283	0.326	0.036	0.356	0.083	0.204
	C		Cc	-0.021	0.015	0.130	0.008	-0.030	-0.027	0.083	-0.087	-0.078	-0.265	-0.046	-0.042	-0.198	-0.179	0.137	-0.215	0.161	0.136	-0.181	-0.293	-0.161	-0.275	-0.082
			Sig.	0.890	0.923	0.384	0.956	0.843	0.857	0.580	0.562	0.601	0.072	0.759	0.777	0.181	0.228	0.357	0.146	0.287	0.369	0.223	0.046	0.279	0.061	0.585
	FAZ	Area	Cc	-0.051	-0.205	-0.245	-0.152	-0.031	-0.125	-0.206	-0.037	0.043	0.136	-0.043	-0.110	0.013	0.062	0.030	0.003	0.020	0.041	0.004	0.269	-0.006	0.280	0.174
			Sig.	0.732	0.168	0.097	0.309	0.834	0.403	0.165	0.804	0.772	0.361	0.772	0.461	0.930	0.677	0.842	0.984	0.897	0.788	0.981	0.067	0.971	0.057	0.242
		Ver. D	Cc	0.054	-0.078	-0.175	-0.022	0.054	-0.025	-0.176	0.075	0.132	0.169	0.021	-0.087	0.131	0.122	-0.092	0.093	0.024	0.101	-0.001	0.242	-0.001	0.260	0.160
			Sig	0.719	0.601	0.239	0.882	0.719	0.865	0.237	0.617	0.378	0.255	0.890	0.560	0.379	0.416	0.537	0.532	0.873	0.504	0.993	0.101	0.995	0.078	0.284
		Hor. D	Cc	0.042	-0.022	-0.062	0.003	0.105	0.039	-0.054	0.062	0.119	0.224	0.073	-0.011	0.165	0.128	-0.052	0.128	0.103	0.178	-0.080	0.192	-0.087	0.206	0.188
			Sig.	0.778	0.883	0.678	0.982	0.481	0.795	0.720	0.679	0.427	0.130	0.627	0.943	0.269	0.392	0.730	0.391	0.498	0.238	0.592	0.197	0.561	0.166	0.205
DCP	S		Cc	-0.038	-0.021	-0.034	-0.135	0.079	0.057	-0.051	-0.022	0.073	0.185	0.014	0.021	-0.012	0.053	-0.033	0.029	0.079	0.065	-0.040	0.113	-0.031	0.145	0.136
			Sig.	0.798	0.887	0.822	0.364	0.596	0.702	0.734	0.884	0.626	0.213	0.924	0.887	0.934	0.722	0.826	0.845	0.600	0.666	0.787	0.448	0.834	0.332	0.361

	T		Cc	0.097	-0.041	-0.030	0.047	0.089	-0.019	-0.055	0.118	0.014	0.134	0.022	0.182	0.014	0.169	-0.097	0.145	-0.062	-0.086	0.038	0.107	0.033	0.083	0.061
			Sig.	0.516	0.787	0.841	0.753	0.554	0.898	0.712	0.430	0.925	0.369	0.882	0.220	0.925	0.255	0.518	0.329	0.684	0.570	0.799	0.473	0.825	0.579	0.684
	I		Cc	0.050	0.138	-0.064	0.096	-0.194	0.043	0.025	0.039	0.041	-0.127	-0.022	0.040	-0.150	0.010	0.011	0.052	0.157	0.110	-0.196	-0.027	-0.194	-0.043	0.032
			Sig.	0.740	0.357	0.667	0.521	0.191	0.774	0.866	0.795	0.783	0.396	0.884	0.787	0.316	0.948	0.939	0.730	0.299	0.468	0.186	0.857	0.192	0.774	0.831
	N		Cc	-0.068	-0.039	-0.233	-0.166	-0.003	-0.049	-0.053	-0.007	0.141	0.130	-0.072	0.030	0.017	0.043	-0.040	0.078	-0.027	0.054	-0.021	0.265	-0.024	0.210	-0.141
			Sig.	0.650	0.796	0.115	0.264	0.982	0.746	0.722	0.964	0.345	0.382	0.629	0.841	0.912	0.776	0.791	0.600	0.856	0.724	0.887	0.072	0.875	0.157	0.344
	C		Cc	0.216	0.249	0.271	0.255	0.230	0.297	0.255	0.132	0.230	0.003	0.013	0.286	0.025	0.154	-0.184	0.044	-0.064	-0.044	0.039	-0.464	0.028	-0.482	0.013
			Sig.	0.144	0.092	0.066	0.084	0.121	0.043	0.083	0.377	0.119	0.983	0.929	0.051	0.867	0.303	0.216	0.771	0.672	0.769	0.796	0.001	0.853	0.001	0.931
	FAZ	Area	Cc	-0.199	-0.229	-0.257	-0.259	-0.117	-0.193	-0.289	-0.196	-0.049	0.071	0.006	-0.248	-0.103	-0.060	0.037	-0.017	0.003	-0.031	0.019	0.273	0.007	0.290	-0.039
			Sig.	0.180	0.121	0.081	0.079	0.432	0.193	0.049	0.188	0.743	0.634	0.970	0.092	0.493	0.688	0.807	0.912	0.982	0.839	0.897	0.064	0.965	0.048	0.795
		Ver. D	Cc	-0.161	-0.140	-0.079	-0.132	-0.148	-0.129	-0.109	-0.065	-0.056	0.063	-0.075	-0.139	-0.004	-0.041	0.023	0.042	-0.091	-0.061	0.106	0.345	0.089	0.341	0.074
			Sig	0.279	0.347	0.595	0.376	0.322	0.389	0.467	0.663	0.707	0.673	0.618	0.350	0.978	0.784	0.880	0.778	0.547	0.685	0.478	0.017	0.550	0.019	0.619
		Hor. D	Cc	-0.032	0.045	0.020	-0.033	-0.002	0.065	-0.041	-0.023	0.010	0.239	0.161	0.039	0.042	0.129	-0.110	0.163	0.139	0.057	-0.131	0.220	-0.143	0.210	-0.065
			Sig.	0.831	0.762	0.892	0.824	0.990	0.665	0.785	0.877	0.948	0.106	0.279	0.795	0.779	0.389	0.463	0.273	0.356	0.706	0.381	0.138	0.337	0.156	0.667
CC	S		Cc	0.255	-0.003	-0.212	-0.166	0.264	0.061	-0.237	0.030	0.409	0.333	0.162	-0.057	0.240	0.248	0.107	0.039	-0.272	-0.191	0.290	-0.011	0.292	0.038	0.231
			Sig.	0.083	0.984	0.152	0.265	0.073	0.682	0.109	0.840	0.004	0.022	0.277	0.703	0.104	0.093	0.475	0.793	0.068	0.203	0.048	0.940	0.047	0.797	0.118
	T		Cc	0.049	0.138	0.162	0.070	-0.002	0.110	0.229	0.052	0.141	0.175	0.200	0.182	0.044	0.113	0.051	0.120	-0.067	0.022	0.051	0.325	0.028	0.301	-0.050
			Sig.	0.742	0.354	0.278	0.641	0.992	0.463	0.122	0.729	0.343	0.240	0.178	0.220	0.768	0.450	0.731	0.423	0.660	0.886	0.733	0.026	0.853	0.040	0.738
	I		Cc	0.009	0.059	0.151	0.175	-0.153	-0.086	0.284	0.082	0.067	-0.133	0.135	0.046	-0.035	-0.032	0.028	0.151	0.019	0.000	-0.080	0.186	-0.092	0.176	-0.170
			Sig.	0.954	0.693	0.311	0.239	0.306	0.566	0.053	0.583	0.657	0.374	0.367	0.759	0.817	0.831	0.851	0.312	0.899	0.998	0.594	0.210	0.538	0.237	0.254
	N		Cc	-0.176	-0.107	-0.114	-0.090	-0.185	-0.220	-0.136	-0.159	-0.254	-0.149	-0.071	-0.030	-0.139	-0.095	-0.032	0.011	-0.042	-0.034	-0.010	0.173	-0.028	0.124	-0.168
			Sig.	0.238	0.474	0.446	0.547	0.214	0.138	0.363	0.286	0.085	0.319	0.635	0.843	0.350	0.524	0.830	0.942	0.780	0.824	0.948	0.245	0.853	0.406	0.260
	C		Cc	0.154	0.187	0.218	0.126	0.177	0.217	0.134	0.235	0.026	0.232	0.184	0.253	0.071	0.158	0.075	0.132	0.041	0.138	-0.103	0.164	-0.113	0.133	0.108
			Sig.	0.301	0.209	0.141	0.397	0.233	0.143	0.368	0.112	0.863	0.116	0.215	0.086	0.634	0.288	0.615	0.375	0.788	0.361	0.490	0.270	0.451	0.373	0.469
BCVA			Cc	-0.287	-0.311	-0.125	-0.169	-0.344	-0.293	-0.202	-0.075	-0.148	-0.284	-0.324	-0.203	-0.255	-0.344	0.168	-0.323	-0.356	-0.313	0.367	-0.156	0.367	-0.151	0.124
			Sig.	0.021	0.012	0.321	0.178	0.005	0.18	0.107	0.552	0.239	0.022	0.008	0.104	0.041	0.005	0.181	0.009	0.004	0.012	0.003	0.215	0.003	0.231	0.326
